# AGDF-Net: Attention-Gated and Direction-Field-Optimized Building Instance Extraction Network

**DOI:** 10.3390/s23146349

**Published:** 2023-07-12

**Authors:** Weizhi Liu, Haixin Liu, Chao Liu, Junjie Kong, Can Zhang

**Affiliations:** 1 College of Mining and Geomatics, Hebei University of Engineering, Handan 056038, China; lwz990324@163.com (W.L.); gislhx@hebeu.edu.cn (H.L.); kongjj1996@gmail.com (J.K.); zhangcan990416@163.com (C.Z.); 2 School of Spatial Information and Geomatics Engineering, Anhui University of Science and Technology, Huainan 232001, China

**Keywords:** building extraction, instance segmentation, attention gate, direction field

## Abstract

Building extraction from high-resolution remote sensing images has various applications, such as urban planning and population estimation. However, buildings have intraclass heterogeneity and interclass homogeneity in high-resolution remote sensing images with complex backgrounds, which makes the accurate extraction of building instances challenging and regular building boundaries difficult to maintain. In this paper, an attention-gated and direction-field-optimized building instance extraction network (AGDF-Net) is proposed. Two refinements are presented, including an Attention-Gated Feature Pyramid Network (AG-FPN) and a Direction Field Optimization Module (DFOM), which are used to improve information flow and optimize the mask, respectively. The AG-FPN promotes complementary semantic and detail information by measuring information importance to control the addition of low-level and high-level features. The DFOM predicts the pixel-level direction field of each instance and iteratively corrects the direction field based on the initial segmentation. Experimental results show that the proposed method outperforms the six state-of-the-art instance segmentation methods and three semantic segmentation methods. Specifically, AGDF-Net improves the objective-level metric AP and the pixel-level metric IoU by 1.1%~9.4% and 3.55%~5.06%.

## 1. Introduction

Automatic building extraction from remote sensing images is a research area of interest due to its wide range of applications in urban planning, population estimation, disaster assessment, and other fields [1,2,3,4]. The popularization of high-resolution remote sensing images provides more convenient and detailed data sources for building extraction [5,6]. However, high-resolution remote sensing images bring more accurate building data but also contain a large amount of intrusive background information, which makes it a challenge to extract buildings with variable appearance in complex environments, such as large cities.

Researchers have made considerable efforts in extracting buildings from remote sensing data for decades. Traditional building extraction methods usually use low-level features [6,7,8] such as colour, spectrum, texture, and geometry, or image information combined with auxiliary information, such as elevation, for building extraction [7,8,9,10,11]. These studies focus on the characteristics of buildings under specific conditions. Therefore, their artificially designed rules are poorly generalized for extracting buildings of different appearances in different areas.

In recent years, many researchers have used deep learning techniques driven by large data samples to solve vision tasks. Convolutional neural networks (CNN) are the most typical deep learning approach. CNN-based methods have been continuously proposed, such as the more mature and popular VGG [12], ResNet [13], UNet [14], SegNet [15], and DeepLab series [16,17,18,19]. Due to the advantage of automatically learning discriminative features, CNNs have been widely used in remote sensing image processing, including change detection [20,21], scene recognition [22,23], and land-use classification [24,25]. Transformer [26] is a later-emerged deep learning model that was first proposed for natural language processing (NLP) tasks. Since the global self-attention mechanism of Transformer is also able to help extract large-scale semantic information in vision tasks, the vision transformer (ViT) [27] was proposed to perform image classification by projecting image patches onto sequences. Transformer-based techniques are also rapidly being used for remote sensing images that are not limited to image classification, such as remote sensing object detection [28,29] and semantic segmentation [30,31].

The success of deep learning in remote sensing image processing has led many approaches to consider building extraction from high-resolution remote sensing images as a semantic segmentation task. These semantic segmentation-based methods output a mask of the same size as the original image and achieve results that exceed those of traditional methods, making them the dominant method for building extraction. For example, GMEDN [32] combines a local and global encoder and a distillation decoder to build an encoder–decoder framework for learning global and local features to adequately describe buildings with different shapes and scales. AMUNet [33] is based on UNet and introduces attention blocks and multiple losses to improve the sensitivity and performance of the model, respectively. Similarly, Chen et al. propose a UNet-based approach and combine ensemble learning with image anomaly detection to extract the location of buildings and estimate their number [34]. Xiao at al. integrated the sliding-window (Swin) transformer with CNN into an encoding enhancer to achieve semantic–local feature fusion and help extract buildings. However, it is still challenging for the semantic segmentation-based methods to segment buildings in complex environments due to the interference of various ground objects with similar building appearances. For example, these methods have difficulty distinguishing multiple nearby building individuals.

Instance segmentation can detect and segment all objects in the image simultaneously and obtain instances of buildings. Unlike semantic segmentation, instance segmentation gives instance-level masks to avoid interference between individual buildings and infers the location and scale of individual buildings and other property information. Owing to these advantages, it has been applied in building extraction studies [35,36,37,38,39,40]. Some methods have been designed to improve the effectiveness of instance segmentation, usually based on Mask R-CNN [41] to improve detection or segmentation capabilities. PointRend [42] builds an iterative segmentation-based algorithm to perform point-based segmentation prediction at adaptively selected locations, resulting in smoother and clearer object boundaries. Mask Scoring R-CNN [43] facilitates mask optimization by adding a maskIoU head that enables it to predict the mask of an instance and the corresponding mask quality. The PANet [44] modified the information flow in the feature extraction network to better utilize the precise location information.

Although the above recently proposed methods significantly improve the performance of instance partitioning networks, for building instance segmentation, there are still some challenging problems to be addressed, which we summarize as follows:

1. Contradictions arise from intraclass heterogeneity and interclass homogeneity. The differences in scale, spectrum, texture, and style within building classes make many special buildings difficult to detect in a complex environment. For example, small buildings are easily missed, large buildings cannot be extracted completely, two parts of a building are recognized as separate individuals, and buildings too close to each other are recognized as the same individual.
2. The angular morphology of buildings is difficult to maintain. Unlike other objects, buildings generally have a regular and sharp morphology. Most of the existing instance segmentation methods are based on convolutional neural networks, where the convolutional kernel operates on the neighbourhood of each pixel, which loses accurate detail information at the boundaries. Similarly, the pooling layer in the network exacerbates this deficiency, and the predicted buildings have difficulty maintaining accurate, regular, and clear boundaries.

To overcome the above problems, we propose an instance segmentation network based on an attention gating mechanism and direction field optimization. And the contributions of this paper are summarized as follows:

1. An instance segmentation network is proposed which effectively distinguishes foreground from background and optimizes building contours in complex environments. Compared to previous approaches, it integrates an Attention-Gated Feature Pyramid Network (AG-FPN) and a Direction Field Optimization Module (DFOM) at the feature extraction and segmentation stages.
2. The AG-FPN is designed to introduce a gated attention mechanism to control the flow between bottom-up and top-down information flows in the feature pyramid network to selectively focus on helpful information and suppress disruptive information.
3. The DFOM is deployed on the mask head to predict the instance-level direction field from the feature map, and the expected direction field assigns a motion orientation to each pixel.

## 2. Methods

In this section, we describe the proposed approach in detail. We first outline the structure of AGDF-Net. Then, the proposed AG-FPN, DFOM, and loss function are elaborated.

### 2.1. Overall Architecture

**Figure 1:**
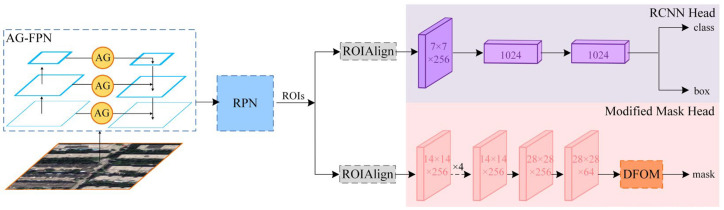
Overview of the structure of AGDF-Net. It has two main improvements, one is that AG-FPN replaces the normal FPN, and the other is that DFOM is deployed in the mask head.

The overall structure of our proposed AGDF-Net is shown in Figure 1. It mainly consists of AG-FPN, RPN, RoIAlign module, cassette head, and modified mask head. Images are first fed to the backbone network of the AG-FPN to obtain feature maps (C2, C3, C4, C5). Then, the AGs measure their regions containing useful and interfering information and control their summation with higher-level multiscale feature maps (P2, P3, P4, P5). Subsequently, the region suggestion network (RPN) generates the region of interest. The box head performs the regression on the size and coordinates of the bounding box and classifies the objects. The mask head is in charge of segmentation within the boxed region. In particular, we modified the original mask head and deployed the DFOM at the tail to optimize the initial segmentation.

### 2.2. Attention-Gated Feature Pyramid Network

To promote the complementarity of semantic and detail information in the feature pyramid network (FPN), we propose an Attention-Gated Feature Pyramid Network (AG-FPN) to selectively integrate detail information by emphasizing the useful information and suppressing the useless information in it.

**Figure 2:**
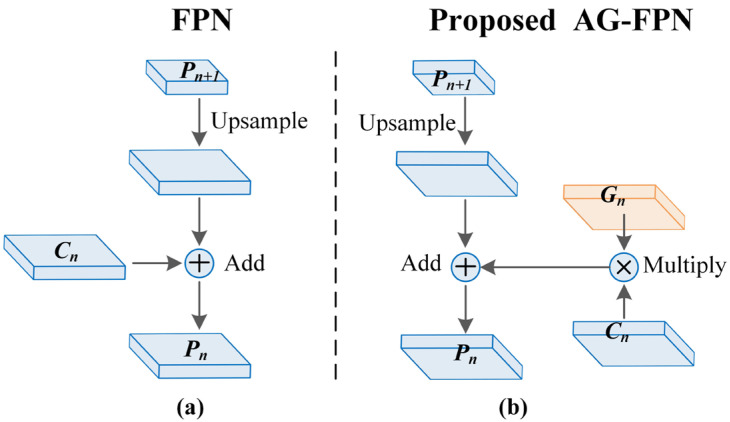
Illustration of the top-down strategies in (**a**) FPN and (**b**) our proposed AG-FPN. The proposed method gives a gating feature map by measuring the importance of information and is used to control the addition of information from the lower-level feature map to the high-level feature map.

Existing instance segmentation methods mostly employ feature pyramid networks (FPN) in the feature extraction stage. FPN first utilizes a backbone network such as ResNet [13] to downsample the input image and obtain feature maps (*C_2_*, *C_3_*, *C_4_*, *C_5_*), with respective strides of 4, 8, 16, and 32. In this bottom-up process, higher-level feature maps such as *C_5_* contain more abstract and semantic information, but are also of a smaller scale; lower-level feature maps such as *C_2_* contain more details and localization information, but are also of a larger scale. Subsequently, FPN again performs a top-down process to gradually upsample the high-level feature map *C_5_* to obtain new multi-scale feature maps (*P_2_*, *P_3_*, *P_4_*, *P_5_*) to gradually restore detail information. The goal of FPN is to fully exploit the semantic and detailed information on each feature map, the former beneficial for classification, and the latter for precise localization. Therefore, FPN uses a residual connection to combine semantic and detail features, as indicated by the arrows in Figure 2a. Specifically, the small-scale feature map of *P_n+1_*, containing richer spatial structure and semantic information, is upsampled and directly added to the large-scale input feature map of *C_n_* to obtain the new *P_n_* feature map.

**Figure 3:**
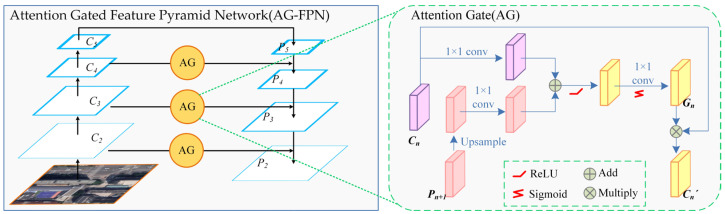
Schematic of AG-FPN.

Nevertheless, this process does not selectively fuse information in *C_n_*, which may introduce useless or intrusive information to *P_n_*, thus affecting the effectiveness of the extracted multi-scale feature maps. The basic idea of the attention mechanism is to enable the system to ignore irrelevant information and focus on the key parts. Therefore, we introduce the gated attention (AG) mechanism to control the information flow in the residual connection of FPN, as shown in Figure 2b. The specific structure of our proposed AG-FPN is shown in Figure 3, where AG first combines the lower-level feature map *C_n_* and the higher-level feature map *P_n−1_* to obtain a gating map *G_n_* to selectively emphasize or suppress certain spatial regions. By multiplying the attention coefficients on *G_n_* with the feature map combined with the lower-level information, *C_n_’* is obtained, and this AG-FPN can focus on the features useful for the final prediction of the building region. Specifically, the calculation formula is as follows:

(1) Pn=Pn+1+Gn’=Pn+1+Gn⋅Cn

(2) Gn=Sigmoid(conv(ReLu(conv(up(Pn+1))+conv(Cn))))

where *P_n_* and *P_n+1_* denote the feature maps at different levels in the information flow from the top down. *C_n_* and *C’_n_* represent the original feature map and the gated feature map in the top-down information flow, respectively. *G_n_* denotes the gating feature map derived from *C_n_* and *P_n_*. Sigmoid and ReLU stand for Sigmoid function and linear rectification unit, respectively. Conv and up denote the 1 × 1 convolution and upsampling operations, respectively.

### 2.3. Directional Field Optimization Module

In image segmentation, inter-class fuzziness and intra-class inconsistency are generally present. Specifically for building extraction, intra-class inconsistency can manifest as scale, shape, colour, and texture divergence of buildings, while inter-class fuzziness can manifest as trees, shadows, and roads interfering with buildings. Existing methods usually learn element-level feature representations, ignoring the spatial relationship constraints between pixels, and are prone to forming erroneous and messy boundaries due to inter-class fuzziness and intra-class inconsistency. 

**Figure 4:**
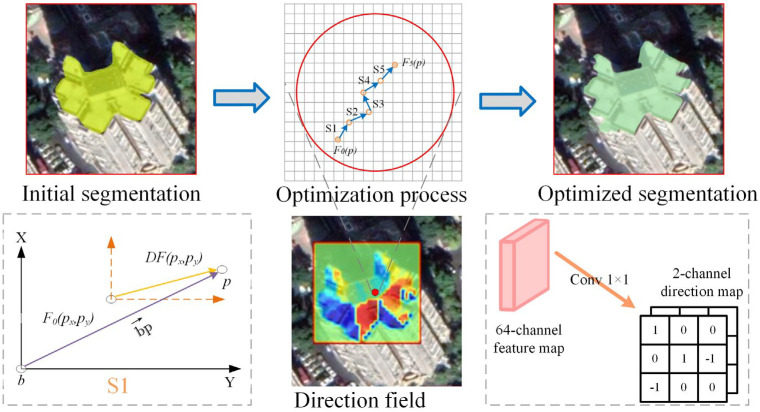
Schematic of DFOM. In DFOM, the 64-channel feature map in the mask head is treated as the initial segmentation, and we perform a 1 × 1 convolution on it to predict the direction field of each pixel (as shown in the lower right corner). The predicted direction field feature map provides a moving direction for each pixel on the initial segmentation, which is used to guide the pixel’s step-by-step flow and to obtain an optimized segmentation. The lower left corner is a schematic of the correction process. $\vec{bp}$ is a directional vector. *F_0_*(*p_x_, p_y_*) denotes the pixel p predicted by *F*_0_(*p*). *DF*(*p_x_, p_y_*) refers to the position of the corresponding pixel *p* in the direction field feature map. *F_0_*(*p_x_, p_y_*) is corrected once through $\vec{bp}$ so that the building boundary is optimized once.

This study modified the mask head of the instance segmentation network and embedded a Directional Field Optimization Module (DFOM) to learn the spatial relationship and semantic association between pixels of each building. This improvement was used to optimize the segmentation of buildings, especially the quality of the boundaries. The workflow of DFOM is shown in Figure 4, and the main tasks can be divided into two parts: direction field prediction and feature optimization. Following the definition in [45], we defined a direction field for each pixel *p*, as shown in Formula (3). In the image domain, for each foreground pixel *p*, i.e., the pixel in the building region, we find the nearest boundary pixel *b* and assigned the unit vector *bp* from *b* to *p* as the direction field of *p*. For background pixels, we set the direction field of these pixels to (0,0). Through the defined directional field, we could establish a connection between the boundary pixels and the building body pixels and indirectly represent the overall shape of the building. To obtain direction field labels, we used the Distance Transform algorithm [46] to compute the ground truth of the direction field according to the mask label. We used 1 × 1 convolution operations to achieve direction field learning and prediction in the network. Specifically, we assigned the 64-channel feature map in the mask head as the initial segmentation map *F_0_*^(^*p*^)^∈*R^64^^×^^H^^×^^W^*. By performing 1 × 1 convolution operations on *F*_0_(*p*), a two-channel predicted direction field $\vec{DF}(p)$∈*R^2^^×^^H^^×^^W^* could be obtained. The defined true direction field and the predicted direction field can be represented as follows:

(3) $$\vec{DF}(p)=\left\{\begin{array}{l} \frac{\vec{bp}}{\left|\vec{bp}\right|}\;\;\;\;\;\;\;\;p\in foreground,\\ (0,0)\;\;\;\;otherwise. \end{array}\right.$$

(4) DFpred=conv(FIS)

where $\vec{DF}(p)$ refers to the direction field of pixel *p*. $\vec{bp}$ represents the vector pointing to *p* from the nearest boundary pixel *b*. *DF_pred_* and *F_IS_* refer to the predicted direction field and initial segmentation.

Once the direction field was predicted, we used a step-by-step iterative optimization algorithm to correct the errors of the initial segmentation map. First, *F_0(p)_* and *DF_(p)_* were gridded to determine their coordinates. The lower left corner in Figure 4 is a schematic of the correction process, $\vec{bp}$ is a directional vector. DF(px,py) is the position of the corresponding pixel *p* in *DF*. *F_0_*(*p_x_, p_y_*) was corrected once through $\vec{bp}$ so that the building boundary was optimized once. Briefly, the improved feature map *F_k_ (p)*∈R*^C×H×W^* was iteratively updated according to the location pointed by $\vec{bp}$ provided by *DF (p)* and was calculated by bilinear interpolation. For *F_0_*(*p*), after a correction process *S1*, a corrected feature map *F_1_*(*p*)∈R*^C×H×W^* could be obtained. The whole process is as follows:

(5) $$\forall p\in \Omega ,F^{k}(p)=F^{\left(k-1\right)}(p_{x}+DF(p_{x})\;,\;p_{y}+DF(p_{y})),\;k\in \{1,2,\ldots N\}$$

After the above *N*-step (*N* = 5 in this study) correction process, we concatenated *F_N_(p)* with *F_0_(p)* and then performed convolution on the concatenated feature maps to predict the final building segmentation.

### 2.4. Loss Function

Following the definition of Mask R-CNN [41], the total loss function combines three sub-loss functions: classification loss $L_{cls}$, bounding box loss $L_{bbox}$, and mask loss $L_{mask}$. In particular, we introduced a sub-loss function $L_{DF}$ for supervised direction field learning into the total loss function. $L_{DF}$ consists of the Euclidean distance and the angular distance between the predicted and true direction fields. The total loss is defined as follows:

(6) $$L_{total}=L_{cls}+L_{bbox}+L_{mask}+L_{DF}$$

where $L_{DF}$ is defined as

(7) $$L_{DF}=\sum_{p\in \Omega }\left||DF(p)-\hat{DF}(p)|{|}_{2}+||\cos^{-1}\langle DF(p),\hat{DF}(p)\rangle |{|}^{2}\right)$$

where DF(p) and $\hat{DF}(p)$ are the direction truth probability of pixel *p* and the predicted DF probability, respectively.

## 3. Experiments and Analysis

### 3.1. Experimental Setup

#### 3.1.1. Dataset

**Figure 5:**
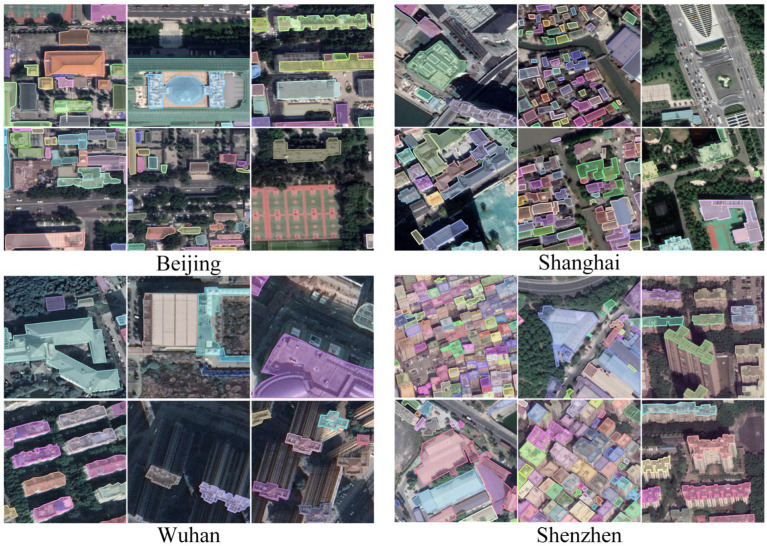
Samples of the dataset of building instances of typical cities in China.

A large number of training samples usually drives and helps validate the effectiveness of deep learning-based building extraction. So, we chose a large dataset named “A dataset of building instances of typical cities in China” [47] for our experiments. The dataset contains a total of 7260 images, of which 5985 were used for training and 1275 for testing. Each image in the dataset has a size of 500 × 500 pixels and a ground resolution of 0.29 m. Examples of the images from each city are shown in Figure 5.

We believe that this dataset allows us to fully test the effectiveness and generalization of models by sampling large building instances of different scales, shapes, textures, and styles across regions.

#### 3.1.2. Implementation Details

All experiments were conducted in a Pytorch environment and with an NVIDIA Tesla V100 GPU, using ResNet-50-FPN as the backbone of all networks in the experiments. For training, we used an SGD optimizer with an initial learning rate of 0.0025, a batch size of 4, a total of 36 epochs, weight decay and momentum settings of 0.0001 and 0.9, and 20% of the training set was randomly selected for validation in each epoch. Data augmentation methods were used during training, including random flipping and rotation, adding noise points, blurring, and gray-value transformation. These methods can increase the data diversity to alleviate overfitting.

#### 3.1.3. Evaluation Metrics

This study uses mean average precision (AP), a standard MS COCO metric, including AP under multiple intersection over union (IoU) thresholds, to evaluate the building instance segmentation task. The equation for IoU is given below:

(8) $$\mathrm{IoU}=\frac{area(M_{{}_{p}}\cap M_{g})}{area(M_{{}_{p}}\cup M_{g})}$$

where *M_p_* and *M_g_* are the predicted mask and its corresponding ground truth, respectively.

Specifically, the AP is calculated at 10 IoU overlap thresholds from 0.50 to 0.95 with a step size of 0.05. The equation is as follows:

(9) $$AP=\frac{AP_{0.5}+AP_{0.55}+\ldots +AP_{0.95}}{10}$$

### 3.2. Comparison Experiments

#### 3.2.1. Qualitative Analysis

To verify the effectiveness of the proposed AGDF-Net, we conducted comparison experiments with several start-of-the-art instance segmentation methods, including Mask R-CNN [41], PointRend [42], MS R-CNN [43], SOLOv2 [48], YOLACT [49], and HTC [50].

**Figure 6:**
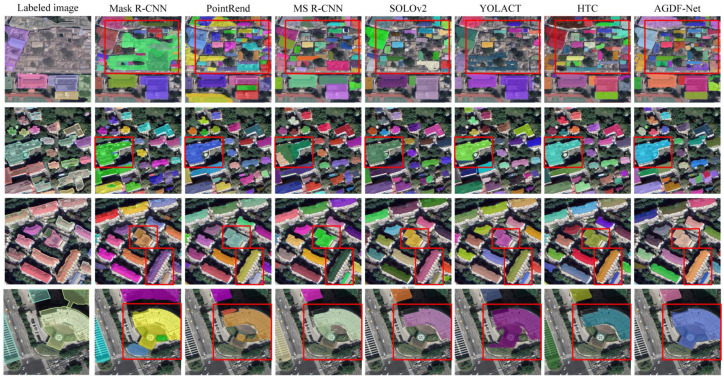
Visualization of the results of the comparison experiments.

Figure 6 shows some visual samples of the results of the comparison experiments. In Figure 6, the different rows represent the different input images, and the different columns denote the methods designed in the experiments. The red boxes marked in Figure 6 indicate some regions where the buildings were not well segmented. 

As we can see, Mask R-CNN may incorrectly detect some false buildings (e.g., roads, shadows, and trees) and miss some smaller buildings in the presence of complex background interference. This is due to the fact that the FPN used in Mask R-CNN adopts a simple residual concatenation, which directly adds features from the bottom-up information flow to those from the top-down information flow. This leaves the information flow of the network vulnerable to a large amount of noise and unnecessary interference information. PointRend refines the segmentation predictions for points selected from specific regions of the initial segmentation iteratively, improving the quality of the segmentation of building contours. However, this refinement is based entirely on the initial segmentation and does not improve the information flow of the feature extraction network, limiting its effectiveness in complex contexts. MS R-CNN adds a mask loU branch to Mask R-CNN for learning the quality of the predicted instance masks and prioritizes the masks with higher mask confidence, aiming to improve the quality of the masks. However, it is difficult to establish an optimal rule to balance the learning prediction of the mask and its quality, making the final mask quality unsatisfactory. As a result, MS R-CNN did not demonstrate significant excellence in our experiments. SOLOv2 introduces a new scheme to dynamically segment objects based on location without the need to determine the object box, making it a single-stage instance segmentation method. This allows it to produce high-resolution masks feature representations, yielding more fine building contours compared to Mask R-CNN. However, its ability to recognize buildings in complex backgrounds is still not improved. Another single-stage model, YOLACT, does not work as well as other ordinary instance segmentation networks in terms of accuracy when extracting a diverse variety of buildings on a large scale. HTC employs a hybrid cascade strategy to combine segmentation and detection tasks in a multi-stage process and adds additional semantic segmentation feature inputs to the segmentation branch. These advantages allow HTC to fully complement the features and strengthen the association between object detection and semantic segmentation tasks. Moreover, its ability to distinguish between foreground and background is improved, making it better at extracting building instances in complex environments. Our experimental results for the AGDF-Net are shown in the last column of Figure 6, achieving better performance in these cases. This is based on the ability of the proposed AG-FPN to measure the importance of information when performing bottom-up and top-down information flow interactions. The improvement with the FPN means that it can better combine spatial structure and semantic information in feature maps at different levels, enhancing key information and complementing information that is lacking for the final feature map, thus reducing missed detections and false recognitions. Furthermore, thanks to the proposed DFOM, the modified mask head can model the spatial relationship between building pixels to control the overall building shape, obtaining more regular and accurate masks.

Specifically, the first row of images in Figure 6 shows a dense urban neighbourhood scene, limited by the quality of the annotation, where the dataset provides labels that directly annotate a large number of individual, relatively small houses as a whole. In this case, the compared methods suffer from the interference of trees and the excessive proximity between houses, missing many buildings or identifying them as a whole. In contrast, AGDF-Net obtained the most complete individuals from the buildings. The images in the second row contain a large building with a terrace of similar colour to the ground; most methods either recognize the terrace as background or fail to recognize the building as a whole. The proposed method effectively overcomes this shortcoming and yields the best-fitting mask for this building instance. The building in the lower right corner of each image in the third row has a jagged outline that prevents most methods from capturing its true contours, and AGDF-Net gives the most realistic mask boundary. The fourth row of Figure 6 shows a large building with a unique style, which partly contains artificial patterns and greenery, resulting in most methods segmenting only its main body. However, AGDF-Net completely extracts it and maintains a clear boundary.

These results visually demonstrate that the proposed AGDF-Net can outperform the performance of Mask R-CNN, PointRend, MS R-CNN, SOLOv2, YOLACT, and HTC on the dataset of the building instances of typical cities in China.

#### 3.2.2. Quantitative Analysis

To better measure the performance of each method, we evaluated them quantitatively, as shown in Table 1. Since most of the methods in the experiments are based on Mask R-CNN improvements or inspired by Mask R-CNN, we used Mask R-CNN as the baseline method for comparison.

**Figure 7:**
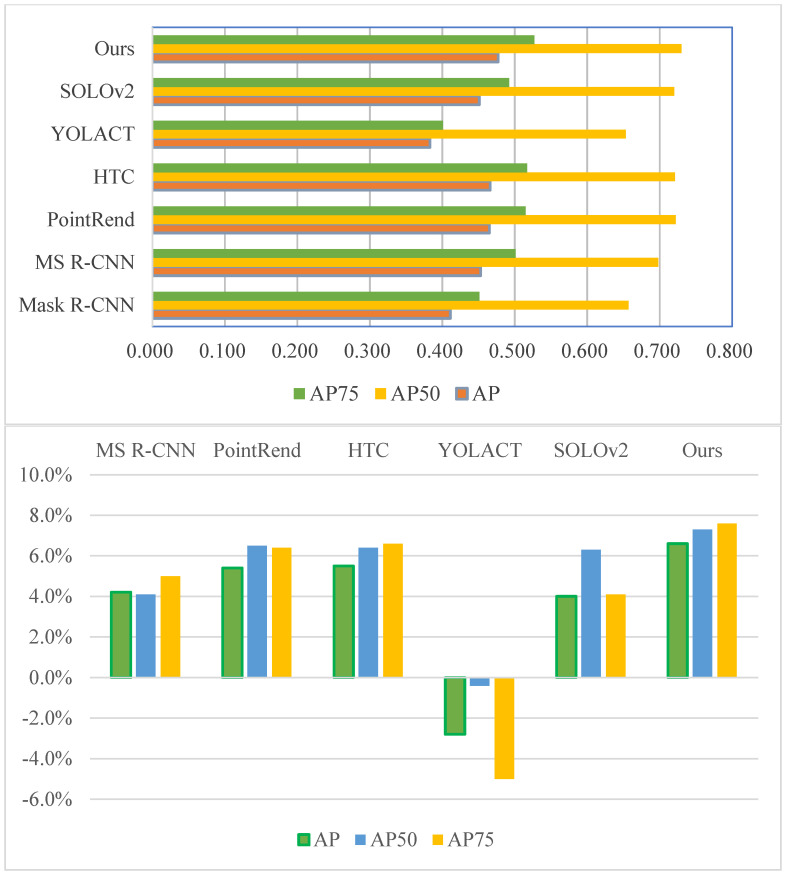
Bar chart comparing the accuracy of the different methods (**top**) and their accuracy improvement (**bottom**) relative to the baseline method (Mask R-CNN).

As seen in Table 1, the proposed AGDF-Net ranks first in AP regardless of the IoU threshold used, which can be visualized in the histogram on the left side of Figure 7. AGDF-Net improves AP, AP_50_, and AP_75_ compared to the baseline by 6.6%, 7.3%, and 7.6%, respectively. Compared to the method ranked second in accuracy, AGDF-Net improves AP, AP_50_, and AP_75_ by 1.1%, 0.8%, and 1.0%, respectively, which is well illustrated in the bar chart on the right side of Figure 7. This indicates that the modifications of the proposed AGDF-Net concerning Mask R-CNN result in a significant performance improvement and outperform the six state-of-the-art generic instance segmentation algorithms. In general, most of the six state-of-the-art methods more or less improved from the baseline for the building instance segmentation task. Among them, the most significant increase is in HTC, which confirms the effectiveness and great potential of the cascade strategy and multi-stage processing algorithm it adopts. It is worth noting that YOLACT has a noticeable accuracy loss compared to the baseline, which may be due to its simplified algorithm process that makes it difficult to cope with building extraction in complex environments.

The quantitative evaluation results further confirm the effectiveness of the proposed AGDF-Net and its superiority over the six state-of-the-art methods for the building instance extraction task.

### 3.3. Ablation Study

To investigate the effectiveness of the proposed AG-FPN and DFOM, we performed ablation experiments.

#### 3.3.1. Ablation for AG-FPN

**Figure 8:**
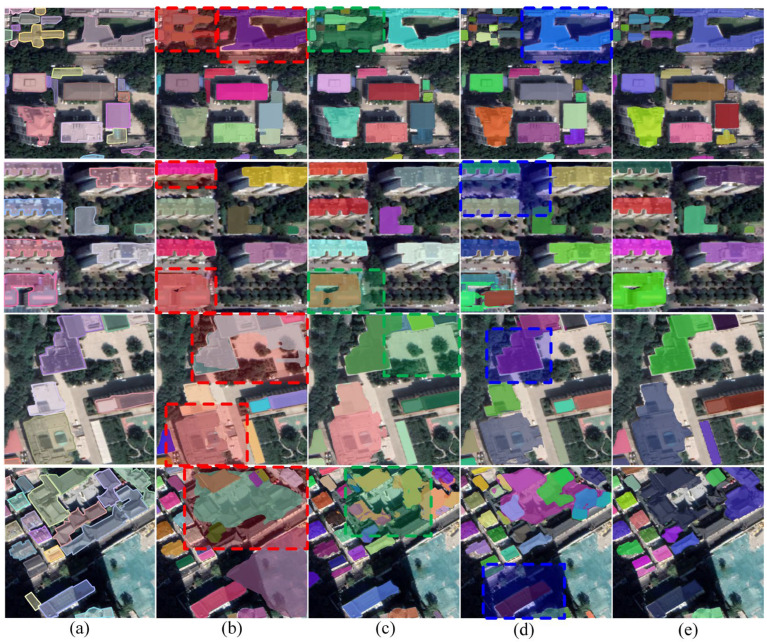
Visualization of the results of the ablation experiments: (**a**) labelled image, (**b**) baseline, (**c**) baseline + AG-FPN, (**d**) baseline + DFOM, (**e**) proposed AGDF-Net. The red dashed box represents the defective area of the baseline method, the green dashed box represents the area improved by AG-FPN, and the blue dashed box represents the area refined by DFOM.

Table 2 and Figure 8 show the results of the ablation evaluation of AG-FPN and DFOM on the dataset of building instances of typical cities in China. Table 2 demonstrates that the application of AG-FPN enhances all metrics, improving AP, AP_50_, and AP_75_ by 3.2%, 5.7%, and 3.0%, respectively. In Figure 8, some small buildings missed by the baseline method can be recognized by AG-FPN, and background interferences such as a ground of a similar colour to the buildings, trees, and the shadows of neighbouring buildings can be suppressed by AG-FPN. The representative area framed by the green dashed line shows well the enhancement of the baseline method by AG-FPN. This is because AG-FPN selectively emphasizes or suppresses information by measuring the importance of information, allowing it to identify more true buildings and fewer false buildings. These results show that AG-FPN can extract key features and reduce interference in building instance extraction.

#### 3.3.2. Ablation for DFOM

The ablation of DFOM was also performed on the dataset of building instances of typical cities in China. Figure 8b,d show the visual results with and without DFOM, respectively. We can see that the model without DFOM is more likely to obtain inaccurate segmentation results for buildings with complex contours. Moreover, the regular boundaries of the buildings are more difficult to maintain for the model without DFOM. These results suggest that DFOM plays a role in optimizing building boundaries. And Figure 8e shows that the optimization role of DFOM can be further enhanced when AG-FPNs are applied, which indicates that the two modifications we proposed for the building are compatible. Table 2 demonstrates that the AP is improved when DFOM is applied. In particular, AP_75_ receives the most significant enhancement due to the boundary optimization that achieves higher-quality segmentation results. The quantitative evaluation of DFOM’s ablation results agrees with those hinted at by the qualitative results, demonstrating DFOM’s effectiveness.

## 4. Discussion

Although AGDF-Net is an instance segmentation method, we also compared it with state-of-the-art semantic segmentation methods including UNet [14], DeepLabv3+ [19], and BRRNet [51] to explore the advantages of the proposed method for the building extraction task.

To compare under the same criteria, we transformed the building extraction results of AGDF-Net into binary masks and evaluated them based on the following pixel-level metrics:

(10) $$\mathrm{Precision}=\frac{TP}{TP+FP}$$

(11) $$\mathrm{Recall}=\frac{TP}{TP+FN}$$

(12) $$\mathrm{IoU}=\frac{TP}{TP+FP+FN}$$

(13) $$F1-\mathrm{Score}=\frac{2\times \mathrm{Precision}\times \mathrm{Recall}}{\mathrm{Precision}+\mathrm{Recall}}$$

(14) $$\mathrm{OA}=\frac{TP+TN}{TP+TN+FP+FN}$$

where TP, TN, FP, and FN represent the true positives, true negatives, false positives, and false negatives, respectively. IoU and OA mean the intersection over union and overall accuracy, respectively.

**Figure 9:**
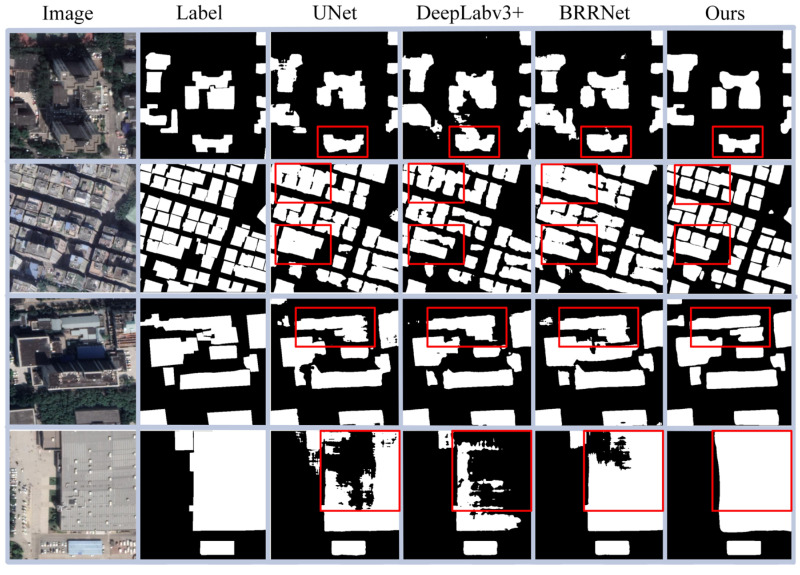
Visualization results of comparison with state-of-the-art semantic segmentation methods.

As shown in Figure 9, our method produces more satisfactory binary masks than semantic segmentation. Specifically, our method not only segmented more accurate boundaries, as shown in the first row, but also maintained the independence between buildings well, see the second row. Moreover, the images in the third row show that the three semantic segmentation methods are generally prone to producing broken boundaries, while the proposed method obtains smoother boundaries. In addition, the proposed method can better detect large buildings. In contrast, the compared semantic segmentation methods tend to generate hollow segmentation results, see the fourth row of Figure 9. The pixel-level quantitative evaluation results in Table 3 also show that the proposed method outperforms the three advanced semantic segmentation methods in most metrics.

The reasons for this advantage, we believe, can be summarized as two main aspects. First, the instance segmentation method segments the building based on the detected regions of building individuals rather than classifying each pixel in the whole image as the semantic segmentation method does. The semantic segmentation method works in a way that tends to lead to a lack of connection and constraint between individual pixels. In contrast, the instance segmentation method can segment within the constraint of the building area. Secondly, AG-FPN and DFOM enhance the model’s ability to better distinguish foreground and background and optimize building boundaries.

## 5. Conclusions

This paper proposes an attention-gated and direction-field-optimized building instance extraction network (AGDF-Net) for accurate and complete building extraction in complex environments while maintaining angular morphology. The proposed method is compared with six state-of-the-art instance segmentation methods on the dataset of building instances of typical cities in China. The qualitative analysis shows that AGDF-Net can better recognize buildings and maintain their boundaries. The qualitative analysis shows that AGDF-Net can achieve an AP of 0.477, which is 1.1%~9.4% ahead of the comparison methods. The ablation experiment explored the effect of the two proposed modules, increasing the AP of the baseline method by 3.2% and 2.1%, respectively. In addition, the discussion section compares the proposed AGDF-Net with state-of-the-art semantic segmentation methods and achieves an IoU of 77.87%, which is 3.55%~5.06% ahead of the comparison methods.

## Figures and Tables

**Table 1:** Comparison of the accuracy of different instance segmentation methods. The best and second-best results are bolded and underlined, respectively.

Methods	AP	AP_50_	AP_75_	AP↑(%)	AP_50_↑(%)	AP_75_↑(%)
Mask R-CNN	0.411	0.657	0.451	-	-	-
MS R-CNN	0.453	0.698	0.501	4.2	4.1	5.0
PointRend	0.465	0.722	0.515	5.4	6.5	6.4
HTC	0.466	0.721	0.517	5.5	6.4	6.6
YOLACT	0.383	0.653	0.401	−2.8	−0.4	−5.0
SOLOv2	0.451	0.720	0.492	4.0	6.3	4.1
Ours	**0.477**	**0.730**	**0.527**	**6.6**	**7.3**	**7.6**

**Table 2:** Quantitative evaluation of ablation experiments.

Methods	Modules		Metrics			Improvements		
	AG-FPN	DFOM	AP	AP_50_	AP_75_	AP↑(%)	AP_50_↑(%)	AP_75_↑(%)
Baseline			0.411	0.657	0.451	-	-	-
Baseline+AG-FPN	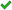		0.443	0.714	0.481	3.2	5.7	3.0
Baseline+DFOM		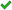	0.432	0.672	0.493	2.1	1.5	4.2
Baseline+AG-FPN+DFOM	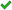	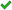	0.477	0.730	0.527	6.6	7.3	7.6

**Table 3:** Comparison of accuracy with semantic segmentation methods. The best and second-best results are indicated in bold and underlined, respectively.

Methods	OA (%)	IoU (%)	F1-Score (%)	Precision (%)	Recall (%)
UNet	92.18	74.32	84.51	84.21	86.24
DeepLabv3+	93.19	72.81	83.47	**86.63**	81.97
BRRNet	92.95	73.24	83.75	82.73	86.39
Ours	**94.22**	**77.87**	**87.14**	84.48	**91.21**

## Data Availability

The data used to support the findings of this study are available from the corresponding author upon request.
